# 2315. Impact of MSSA Surveillance and Decolonization on Invasive Infection in the Neonatal ICU

**DOI:** 10.1093/ofid/ofac492.147

**Published:** 2022-12-15

**Authors:** Patrick J Reich, Geoffrey Ikpeama, Angela Niesen, Caren Liviskie, Christopher McPherson, Stephanie A Fritz

**Affiliations:** Washington University School of Medicine, Saint Louis, MO; St. Louis Children's Hospital, St. Louis, Missouri; St. Louis Children's Hospital, St. Louis, Missouri; St. Louis Children's Hospital, St. Louis, Missouri; St. Louis Children's Hospital, St. Louis, Missouri; Washington University School of Medicine, Saint Louis, MO

## Abstract

**Background:**

Invasive methicillin-susceptible *Staphylococcus aureus* (MSSA) infections are an important cause of morbidity and mortality in neonatal intensive care unit (NICU) infants. MSSA colonization (asymptomatic carriage in the nose, skin, or gut) is a risk factor for subsequent invasive infection (e.g., pneumonia, bone infections, bloodstream infections, etc). Active surveillance programs coupled with decolonization measures for MSSA-colonized infants have been associated with decreased invasive MSSA infection rates in the NICU setting.

**Methods:**

In June 2020, an active surveillance program was implemented to detect MSSA nasal colonization for infants admitted to the St. Louis Children’s Hospital NICU, a 140-bed quaternary NICU. MSSA-colonized infants were decolonized with a 7 day course of intranasal mupirocin twice daily plus topical chlorhexidine baths on days 1, 4, and 7 (for infants greater than 30 weeks postmenstrual age). MSSA bloodstream infection (BSI) rates were followed quarterly pre- (September 2018-May 2020) and post- (June 2020-March 2022) implementation of MSSA surveillance and decolonization. Mean MSSA BSI rates pre- and post-implementation were compared via paired T-test. The number of infants treated with mupirocin was calculated pre- and post-implementation.

**Results:**

After implementing MSSA surveillance and decolonization there was no significant change in the mean MSSA BSI rate (**Figure**): 0.22 per 1000 patient-days pre-implementation, 0.16 per 1000 patient-days post-implementation (p=0.37). Since initiating MSSA decolonization in June 2020, the number of NICU infants exposed to mupirocin increased nearly fourfold: mean 10 infants treated per month pre-implementation, mean 38 infants treated per month post-implementation.

**Conclusion:**

Implementing an MSSA surveillance and decolonization program in the NICU was not associated with a sustained decrease in invasive MSSA infections. However, mupirocin use in the NICU nearly quadrupled following implementation. Additional investigation is warranted to identify potential contributing factors for unchanged invasive MSSA infection rates despite active surveillance and decolonization (e.g., emerging mupirocin resistance).

**Disclosures:**

**All Authors**: No reported disclosures.

**Figure.**
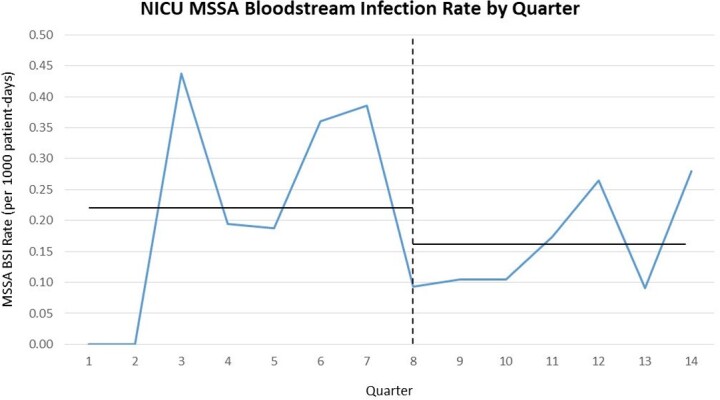
Neonatal Intensive Care Unit (NICU) methicillin-susceptible Staphylococcus aureus (MSSA) bloodstream infection (BSI) rate per 1000 patient-days by quarter from June 2018 (beginning of quarter 1) through March 2022 (end of quarter 14). The dashed vertical line at the beginning of quarter 8 indicates the start of the implementation period. The mean MSSA BSI rates for the pre- and post-implementation periods are indicated by the horizontal lines.

